# fNIRS-based neurofeedback targeting the right dorsolateral prefrontal cortex (dlPFC) in children at familial risk for substance use disorder and/or severe mental illness: a proof-of-concept study from eastern India

**DOI:** 10.3389/fnhum.2026.1936277

**Published:** 2026-09-09

**Authors:** Titli Saha, Poulami Giri, Rajnita Nag, Debosmita Ganguly, Natasha Patel, Ayoleena Roy, Shirshendu Chaudhuri, Kamini Verma, Arghya Pal, Kumari Rina, Sukanto Sarkar, Aniruddha Basu

**Affiliations:** 1 All India Institute of Medical Sciences, Kalyani, India; 2 Indian Institute of Public Health, Hyderabad, India

**Keywords:** DLPFC, executive function, fNIRS, neurofeedback, pediatric vulnerable population, working memory

## Abstract

**Background:**

Children who have first-degree relatives diagnosed with substance use disorders and/or severe mental illnesses show altered prefrontal cortical development and an elevated risk of executive function impairment. fNIRS-based neurofeedback (NF) is a portable, child-tolerant technique for the modulation of cortical hemodynamics, with preliminary evidence of dlPFC-dependent cognitive benefits in adult populations. However, evidence evaluating its efficacy in pediatric at-risk cohorts from the Indian subcontinent is currently lacking.

**Methods:**

This single-arm, uncontrolled proof-of-concept study evaluated the feasibility and tolerability of an eight-session, fNIRS-based NF protocol targeting two right dlPFC channels (Ch10, Ch15) in 11 children (aged 6–12 years), recruited from an inpatient psychiatric unit and an addiction treatment facility. Real-time oxygenated hemoglobin (HbO) feedback was provided via Turbo-Satori BCI. Session-wise HbO amplitude and Ch10↔Ch15 functional connectivity (FC) were extracted for all 11 children. Participants completed pre- and postintervention assessments of executive function. Exploratory Spearman correlations examined the associations between dlPFC HbO modulation and pre–post behavioral changes.

**Results:**

All 11 children completed the protocol without adverse events. The ROI-averaged right dlPFC HbO amplitude showed a positive session-wise increase that did not reach conventional significance (*β* = 0.00003, *t*(950) = 1.90, *p* = 0.058). Individual channel slopes were positive but non-significant after FDR correction. Inter-channel FC increased from session 1 (*r* = 0.21) to session 4 (*r* = 0.41) before declining by session 8 (*r* = 0.18), with no significant linear trend. Behavioral measures showed numerically higher accuracy across tasks, and both the Raven’s CPM and the Stroop task showed shorter reaction times postintervention, but none of the comparisons survived Bonferroni correction. A moderate-to-strong correlation was observed between right dlPFC HbO change and Stroop incongruent accuracy improvement, but it was non-significant (*ρ* = 0.679, *p* = 0.094), with no significant associations for the *N*-back or CPM outcomes.

**Conclusion:**

This eight-session right dlPFC fNIRS–NF protocol in children with elevated familial risk was feasible and well tolerated. It showed a positive, but not statistically significant, increase in HbO amplitude. The connectivity between targeted channels and behavioral scores showed a positive correlation, but it was not statistically significant. A strong-to-medium correlation was observed between right dlPFC HbO change and Stroop incongruent accuracy scores. These findings provide preliminary effect-size estimates to inform the design of future sham-controlled trials in this underrepresented pediatric population.

## 1 Introduction

The dorsolateral prefrontal cortex (dlPFC) is a key region involved in regulating cognitive and behavioral functions, particularly executive function (Friedman and Robbins, 2022). Environmental exposures during critical developmental periods can significantly affect the development of executive function in children (Gluckman et al., 2008; O'Donnell and Meaney, 2017). Children growing up in households affected by parental substance use, severe mental illness, or both are exposed to a range of prenatal, perinatal, and postnatal risk factors—including intrauterine exposure to substances—that have been linked to altered prefrontal structure and function, an increased risk of mental health disorders (Quinn et al., 2017), developmental disabilities (Popova et al., 2017), and downstream cognitive (Warner et al., 2006; Konijnenberg and Melinder, 2011), affective, and behavioral impairments (Jutras-Aswad et al., 2009). As a result, children who are first-degree relatives of individuals with substance use disorders and/or severe mental illness represent a population for whom interventions that support prefrontal-dependent executive function may hold substantial translational value. However, these children remain markedly underrepresented in the neuromodulation literature, particularly outside Western contexts.

Neurofeedback (NF) is a closed-loop training paradigm that allows a participant to learn to self-regulate localized neural activity. The measured brain activity is instantaneously conveyed to the participant through a brain–computer interface (BCI), allowing the individual to exert volitional control through various mental strategies (Barth et al., 2016; Sitaram et al., 2017). Unlike open-loop neuromodulation techniques (e.g., transcranial electrical or magnetic stimulation), NF relies on operant learning principles and the brain’s intrinsic capacity for neuroplasticity and self-regulation. It can thus be defined as a form of biofeedback that provides real-time sensory representation of otherwise unconscious physiological processes (Gaume et al., 2016).

Functional near-infrared spectroscopy (fNIRS) is a noninvasive neuroimaging technique that uses near-infrared light to measure relative changes in cortical blood oxygenation levels, providing insights into localized brain activity (Ferrari and Quaresima, 2012). Compared to functional magnetic resonance imaging (fMRI) and electroencephalography (EEG), fNIRS offers a unique combination of advantages for NF in children as it is portable, relatively low cost, tolerant of moderate motion artifacts, and provides superior spatial localization relative to EEG while avoiding the confinement, noise, and strict immobility requirements of fMRI (Wang et al., 2025). These properties make fNIRS well-suited for school-age children, including those from clinical or at-risk backgrounds.

A growing body of work has applied fNIRS-based NF specifically to the dlPFC, predominantly in adult cohorts. Proof-of-concept and pilot studies have demonstrated that healthy adults can learn, over several sessions, to volitionally upregulate dlPFC hemodynamic activity (Li et al., 2023; Hudak et al., 2017). Several studies have linked successful dlPFC self-regulation to improvements in cognitive performance. For example, a sham-controlled trial in 60 healthy adults found that fNIRS-based NF training of the dlPFC produced significant region-specific increases in dlPFC activation relative to sham feedback, accompanied by significant improvements in spatial working memory and attention (Li et al., 2023). A single-session, sham-controlled study (*N* = 62) combining fNIRS-based NF of the left dlPFC with subsequent fMRI during a working memory task found that the NF group showed increased bilateral dlPFC activity and altered dlPFC–insula functional connectivity during the working memory task (Yang et al., 2024).

In addition to changes in working memory associated with dlPFC self-regulation, fNIRS-based NF has shown positive effects on emotion regulation (Marx et al., 2015), inhibitory control (Hudak et al., 2017; Marx et al., 2015), and management of autism spectrum disorder (Liu et al., 2017), ADHD (Wu et al., 2022; Barth et al., 2021), anxiety, and depression (Kimmig et al., 2019). In pediatric populations specifically, fNIRS-based NF protocols have shown tolerability and preliminary behavioral benefits in children with ADHD disorder (Hudak et al., 2017; Wu et al., 2022) autism spectrum disorder (Liu et al., 2017), and following pediatric stroke (Tetsuka et al., 2023). Overall, fNIRS-based neurofeedback offers a promising approach for both clinical applications and neuroenhancement, with the prefrontal cortex being an especially well-suited target for enhancing executive function.

Despite growing evidence supporting beneficial fNIRS-based NF interventions for executive function impairments, no fNIRS-based NF study has, to our knowledge, been conducted in children who are first-degree relatives of individuals with substance use disorders or severe mental illnesses. This cohort exhibits documented prefrontal neurodevelopmental vulnerabilities, making dlPFC-targeted neuromodulation of particular clinical and translational relevance (Thompson et al., 2009; Parolin et al., 2016).

The majority of fNIRS-based NF studies have been conducted in Western populations. To the best of our knowledge, no prior studies have evaluated fNIRS-based NF in the Indian context. Despite exhibiting unique ethnic and genetic diversity, distinct cortical thickness profiles (Kang et al., 2020), and significant morphometric template deviations across individuals aged 6–60 years compared to the Western templates (Holla et al., 2020), the Indian population remains markedly underrepresented in neuroimaging and neuromodulation research. More neuroimaging and intervention studies are therefore warranted in the Indian population, particularly in children with a history of familial vulnerabilities. This population represents a critical target for intervention, given the well-documented neurocognitive deficits observed in at-risk offspring and the potential for neurofeedback to enhance prefrontal cortical function.

The aim of this proof-of-concept pilot study was to develop a right-dlPFC-targeted, fNIRS-based NF protocol for an at-risk Indian pediatric population and to assess its feasibility and tolerability in these children, while evaluating preliminary improvements in executive function scores associated with dlPFC modulation, to inform further large-scale sham-controlled, community-based research.

## 2 Materials and methods

### 2.1 Study design and setting

The study followed a pre–post, single-arm, proof-of-concept design. All participants were recruited as first-degree relatives of patients admitted to the inpatient unit of the Department (IPD) of Department of Psychiatry of at AIIMS Kalyani, India. The sample size was based on feasibility for a proof-of-concept study, rather than formal power calculations.

### 2.2 Participants

**Figure 1:**
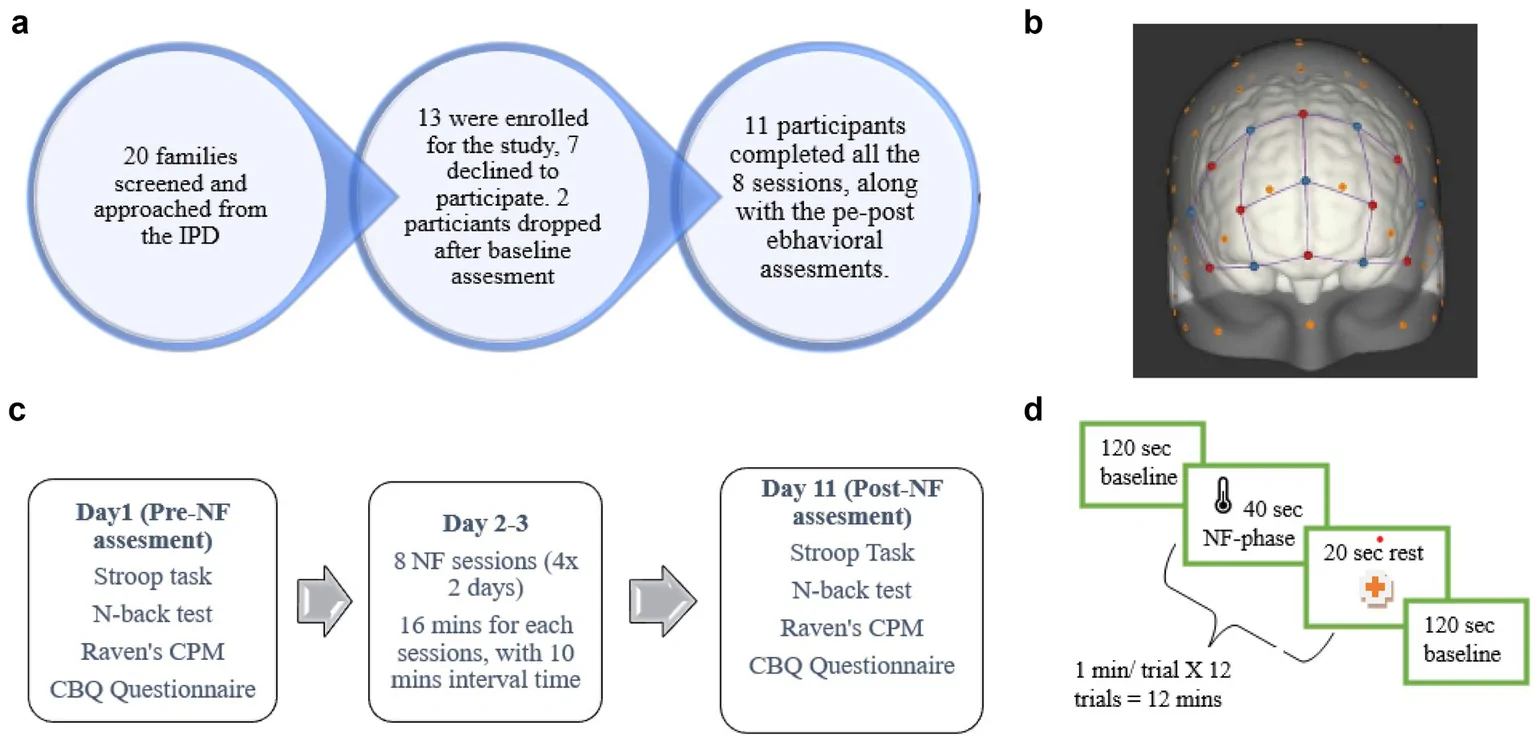
**(a)** Recruitment flow overview. **(b)** Describes the placement of optodes on the brain area. Red denotes the source, and blue denotes the detectors. **(c)** Experimental design for the whole protocol. **(d)** Block design for a single session of the NF training phase.

Twenty families were approached through the inpatient psychiatric department; two withdrew after the baseline cognitive assessment. Seven families declined participation. Eleven children (aged 6–12 years) were enrolled in this preliminary study (Figure 1a). In all 11 families, the affected first-degree relative was a parent, and substance use severity and exposure were documented using the ASSIST (Alcohol, Smoking and Substance Involvement Screening Test) and SADQ (Severity of Alcohol Dependence Questionnaire) scores of the affected parent. Of the eleven children, nine had a parent with substance use disorder, two of whom had comorbid MDD and anxiety. One child had a parent with MDD and anxiety disorder without substance use; and one child had a parent with MDD and psychosis, with no reported parental substance use history. Children were excluded if they had a personal history of severe mental illness or any neurodevelopmental disorder themselves, or if they were unable to understand and follow the instructions required for neurofeedback (NF) training.

All 11 participants completed the eight-session, fNIRS-NF protocol, with channel-level fNIRS data available for at least one of the two right dlPFC target channels (Ch10, Ch15) across sessions. No adverse events, participant discomfort, or invalid trials were reported across the 88 total sessions (11 participants × 8 sessions), and no sessions were rejected as procedurally invalid (e.g., due to incomplete or interrupted procedures). Equipment setup required approximately 3–4 min per participant. All participants completed the pre- and post-intervention behavioral assessment sessions as described in the following sections.

All procedures were conducted with written informed consent from caregivers and assent from child participants, as approved by the Institutional Ethics Committee of AIIMS Kalyani (IEC/AIIMS/Kalyani/Meeting/2022/103). Data were anonymized using de-identified participant codes throughout processing and analysis, and no personal identifiers were associated with the dataset.

### 2.3 Neurofeedback protocol and apparatus

Cortical hemodynamics were recorded using a NIRSport2 continuous-wave fNIRS system (NIRx Medical Technologies, Berlin, Germany) at a sampling rate of 7.81 Hz, using two wavelengths (760 nm and 850 nm) to estimate relative changes in oxygenated HbO and deoxygenated HbR concentration. Sixteen channels (optode pairs) were arranged to cover the bilateral dlPFC, with source (illuminator) and detector optodes (blue) positioned according to a standard montage (Figure 1a). For the present analyses, two channels overlying the right dlPFC were of primary interest: Ch10 (source–detector pair S4–D5) and Ch15 (source–detector pair S6–D5). Coordinates in the probe registration file confirmed right-hemispheric localization for both channels: Ch10 (*x* = +14.8, *y* = 63.3, *z* = 62.2 mm) and Ch15 (*x* = +33.1, *y* = 71.8, *z* = 38.4 mm). A positive *x*-value denotes the right hemisphere, consistent with the intended right dlPFC target region. The two target channels (Ch10, Ch15) were fixed across all study participants across all eight sessions. These channels were selected prior to the study using a Stroop-based localizer task administered to three independent, age-matched children who did not otherwise participate in the study. These two channels, showing the highest task-evoked activation in this independent localizer sample, were designated as the fixed right dlPFC targets for all subsequent NF sessions. This procedure was independent of the outcome measures collected from the 11 study participants.

The NF intervention consisted of eight sessions delivered over two consecutive days (four sessions per day, with a rest interval between sessions), as illustrated in Figure 1c. Each session began with a 120-s baseline period, during which a fixation cross was displayed, followed by a 40-s NF phase and a 20-s rest period (Figure 1d). Each session lasted approximately 16 min, excluding equipment setup. During the NF phase, participants viewed a real-time “thermometer” display reflecting real-time normalized HbO activity in the target channel and were instructed to keep the thermometer bar above a specified threshold. No explicit cognitive strategy was imposed; children were informed prior to the first session that strategies such as mental arithmetic, recalling words or names, or visualizing spatial objects might help raise the thermometer, but they were free to discover their own approach, following previous protocols (Kohl et al., 2020). During the intertrial rest period, a small dot replaced the thermometer to signal the absence of feedback, and children were instructed to allow their minds to wander and to rest to limit mental and physical fatigue. When asked which strategy they had used, nine of the eleven children reported recalling happy memories, familiar places, and test questions as their primary strategy, and two reported mentally solving a Rubik’s Cube; this heterogeneity in self-regulation strategies represents a plausible contributor to the individual variability observed in the HbO trajectories.

Real-time signal processing and feedback were implemented using Turbo-Satori, a dedicated brain–computer interface (BCI) software by Brain Innovation, Germany (Lührs and Goebel, 2017). Raw optical density signals from these target channels were converted to concentration changes and z-scored relative to baseline, and the processed signal was presented to participants as a visual thermometer bar, reflecting relative increases or decreases in HbO concentration. Because the aim of this study was to facilitate volitional upregulation of localized hemodynamics, only the HbO signal was included in the neurofeedback loop.

### 2.4 Behavioral assessments

Three executive function tasks were administered immediately prior to the first NF session (Day 1, pre-intervention) and approximately 7 days after the eighth NF session (Day 11, post-intervention; Figure 1c). The assessments included:

1. Stroop Task: A neuropsychological tool used to measure attention, cognitive flexibility, and inhibitory control (Stroop, 1935). Accuracy and RT were recorded separately for congruent and incongruent trials. The incongruent trials are most strongly associated with prefrontal cognitive control; incongruent-trial accuracy and RT were designated as primary Stroop outcomes.
2. *N*-Back Task: A cognitive task used to assess working memory and attentional control (Kirchner, 1958). Children completed 0-, 1-, and 2-back conditions. Given that the 2-back condition is most consistently associated with dlPFC engagement, the 2-back condition (accuracy and RT) was selected as the primary *N*-back outcome for the brain–behavior correlational analyses.
3. Raven’s Coloured Progressive Matrices (CPM): An assessment tool used to measure nonverbal abstract reasoning and fluid intelligence (Raven and Raven, 2003). The primary outcomes evaluated for analysis were the accuracy and mean response time (RT).

In addition to the cognitive tasks, the Children’s Behavior Questionnaire (CBQ) was administered to caregivers at both time points as part of the parent protocol to characterize child temperament along the broad dimensions of negative affectivity, surgency/extraversion, and effortful control (Rothbart et al., 2001). CBQ outcomes are not the focus of the present report and will be reported separately.

### 2.5 fNIRS signal processing and outcome extraction

Raw fNIRS data were preprocessed using a structured, automated pipeline implemented in MATLAB (MathWorks, version R2025, Natick, MA) using the Homer3 toolbox (version 1.87.0) (Huppert et al., 2009). Raw light intensity data were converted to optical density (OD) and then to relative changes in oxygenated HbO and deoxygenated HbR hemoglobin concentration using the modified Beer–Lambert law, with a differential pathlength factor (DPF) of 6.0 and a source-detector separation of 3.0 cm. Motion artifacts in the resulting HbO time series were then corrected using the temporal derivative distribution repair (TDDR) algorithm (Fishburn et al., 2019), which calculates the temporal derivative of the signal to detect and correct baseline shifts and rapid motion-induced spikes. Motion-corrected signals were bandpass filtered (0.01–0.09 Hz, third-order zero-phase Butterworth filter) to remove slow drift and high-frequency physiological noise, and subsequently linearly detrended. Channels with a coefficient of variation exceeding 7.5% in the raw intensity signal were excluded from analysis as indicative of poor optode–scalp coupling, and channels with a signal-to-noise ratio below 2 were similarly rejected. The primary channels of interest, Ch10 and Ch15 remained well within acceptable thresholds across all the sessions and trials, requiring no channel-level exclusion.

#### 2.5.1 Signal preprocessing and neurofeedback learning analysis

To quantify neurofeedback learning across the eight training sessions, session-wise hemodynamic changes were extracted from the two target channels, Ch10 (S4–D5) and Ch15 (S6–D5), both located over the right dlPFC. Trial-level HbO amplitude was computed using a block-average, baseline-subtraction approach. For each trial, the mean HbO concentration during the 40-s NF and 20-s rest epochs was compared against the mean concentration during the preceding 5-s baseline window. The differences were averaged across all valid trials within each session for each channel, yielding a single per-session, per-channel mean HbO amplitude for each participant. A session-level regulation score was calculated as the mean of the trial-averaged NF amplitude minus the trial-averaged rest amplitude over the two right dlPFC channels (Ch10, Ch15). These oxygenated hemoglobin signal (ΔHbO) values were aggregated at the participant level and then averaged across all participants to produce a group learning trajectory. A composite dlPFC learning index was defined as the average ΔHbO of Ch10 and Ch15 for each session across the entire intervention period. A linear mixed-effects model (LMM) for regression analysis was applied to estimate the direction and magnitude of learning across sessions. Each row represents one trial nested within session nested within participant. Session and Trial were entered as fixed effects, and participant identity (Subject) was modeled as a random intercept; this structure reflects the full trial-level dataset (12 trials × 8 sessions × 11 participants). The regulation-score analysis used a trial-level dataset (maximum 12 trials × 8 sessions × 11 participants = 1,056 observations). A trial was retained only if both its NF and rest windows contained at least 10 recorded samples (≥1.28 s at the 7.81 Hz sampling rate). Trials whose windows fell outside the recorded session duration, such as trials timed near the end of a session that ran short, did not meet this criterion and were excluded prior to model fitting. This is a data-completeness check operated independently of the channel-level signal-quality exclusion criteria described in Section 2.5. After this filtering, 953 trial-level observations remained, yielding the reported denominator degrees of freedom for the Session fixed effect [*t*(950)]. The model yielded an unstandardized slope (*β*), test statistic, and *p*-value for each channel. Here, *β* denotes the fixed-effect regression coefficient for Session, quantifying the average change in HbO amplitude per unit increase in Session (from one session to the next), assuming a linear trajectory across the eight sessions. For the single-channel analyses, *p*-values were corrected for multiple comparisons using the false discovery rate (FDR) procedure (p_FDR).

LMMModel=Regulation score~Session+Trial+(1∣Subject)

#### 2.5.2 Functional connectivity analysis

Functional connectivity between the two right dlPFC channels, Ch10 and Ch15, was explored as a neural outcome during the NF sessions. For each session, Pearson correlation coefficients (*r*) were calculated from the preprocessed HbO time series for the 40-s NF window and the 20-s rest window. Each trial-level correlation was Fisher *z*-transformed, and values were averaged across the 12 trials within a session to yield one NF-condition and one rest-condition FC value per participant per session.

To test for systematic variation in connectivity across training, the NF-condition FC trajectory across the eight sessions was modeled using a linear mixed-effects model [FC ~ Session + (1 | Subject)], which accounted for participant-level repeated measures. To illustrate the time course of the Ch10↔Ch15 coupling pattern, group-mean FC matrices were generated for representative early (Session 1), mid-training (Session 4), and late (Session 8) sessions.

### 2.6 Statistical analysis

For pre- and post-intervention behavioral analyses, behavioral outcomes from *n* = 11 participants (Raven’s CPM raw score and mean RT; *N*-back accuracy and RT across three levels; Stroop incongruent accuracy and RT) were assessed for normality using the Shapiro–Wilk test. Because several distributions departed from normality, the nonparametric Wilcoxon signed-rank test was used as the primary pre–post comparison for all behavioral outcomes, with the rank biserial correlation (*r*) reported as the effect size. Statistical analyses were performed using MATLAB R2025. To account for multiple comparisons, Bonferroni-corrected *p*-values (p_corr) were computed within each task. Two comparisons for CPM (*α* = 0.025), six comparisons for the N-back task across the three levels (*α* = 0.0083), and four comparisons for the Stroop task across the congruent and incongruent conditions (*α* = 0.0125).

For exploratory brain–behavior transfer, hemodynamic changes of the targeted right dlPFC channel were measured as the difference in mean task-evoked HbO (ΔHbO) concentration between post-training and pre-training assessment blocks. Spearman rank correlations were used to examine associations between ΔHbO and pre–post behavioral changes in Stroop incongruent accuracy, *N*-back 2-back accuracy, *N*-back 2-back reaction time, and Raven’s CPM accuracy.

## 3 Results

### 3.1 Sample characteristics and feasibility

All 11 children completed the full eight-session, fNIRS–NF protocol without adverse events, yielding channel-level fNIRS data for both the target channels (Ch10, Ch15) at every session. This full sample (*n* = 11) was used for the neurofeedback acquisition analysis. All 11 participants completed matched pre- and post-intervention behavioral assessments across all three cognitive tasks (*N*-back, Stroop, and Raven’s CPM).

### 3.2 Primary neural outcome: neurofeedback learning across eight sessions

**Figure 2:**
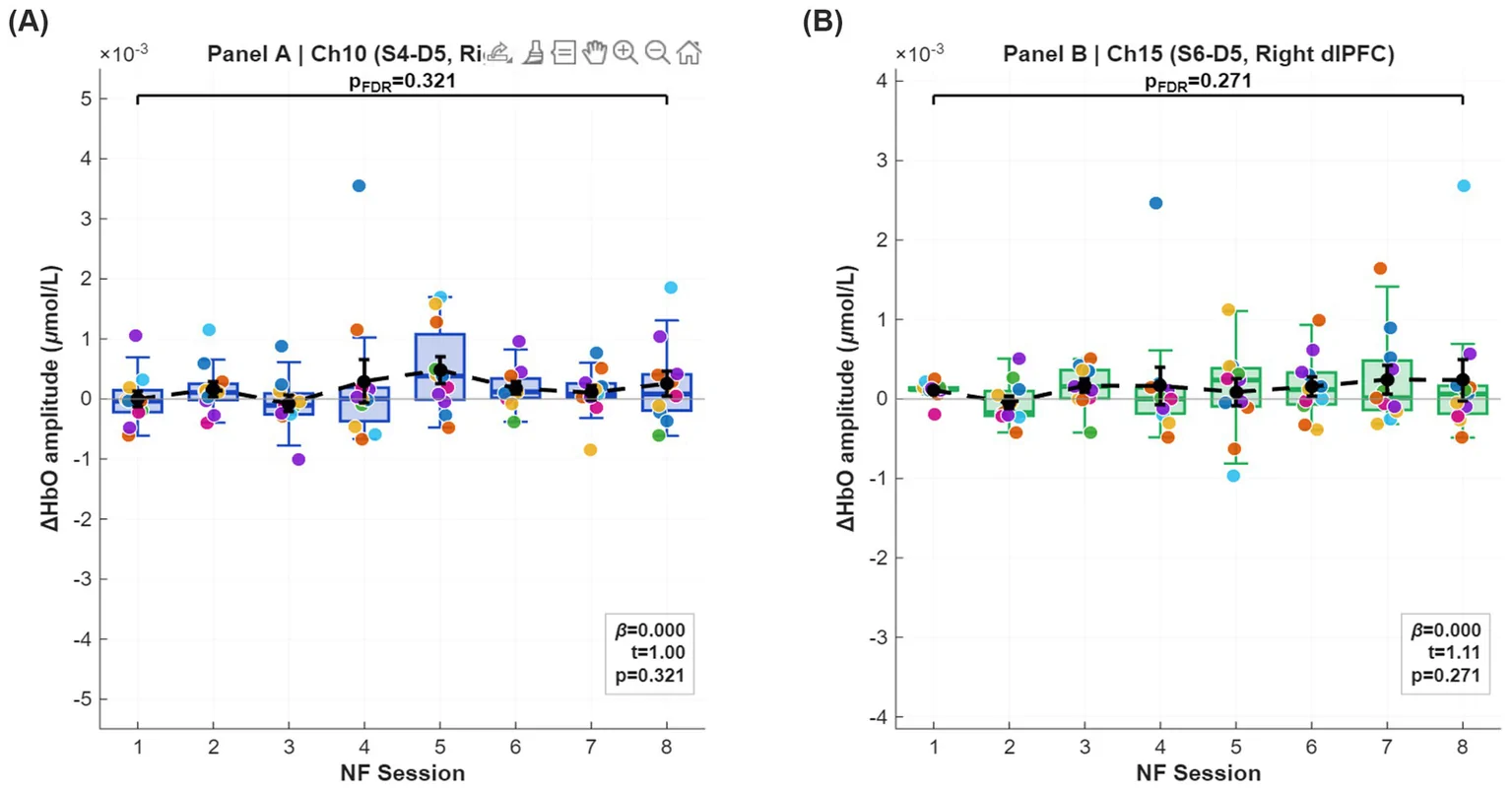
dlPFC HbO upregulation across the eight fNIRS–NF sessions (*n* = 11). **(A)** Ch10 (S4–D5, right dlPFC), *β* = 0.000, *t* = 1.00, *pFD_R_* = 0.321. **(B)** Ch15 (S6–D5, right dlPFC), *β* = 0.000, *t* = 1.11, pFD*_R_* = 0.271.

Across the eight NF sessions, the group-mean ΔHbO in both dlPFC target channels showed a positive trajectory (Figure 2). For Ch15 (S6–D5), ΔHbO increased from approximately 0.0 × 10^−4^ mM·mm at Session 1 to a peak of approximately 4.8 × 10^−4^ mM mm at Session 5, before declining by Session 8. For Ch10 (S4–D5), ΔHbO exhibited an early upward trajectory, with a broad peak across Sessions 4–5 and a slight increase at Session 8. Although neither linear trend reached significance after FDR correction, both dlPFC channels ended the intervention above their early-session baseline levels. Overall, the NF dataset demonstrated a trend toward progressive dlPFC upregulation during training.

**Figure 3:**
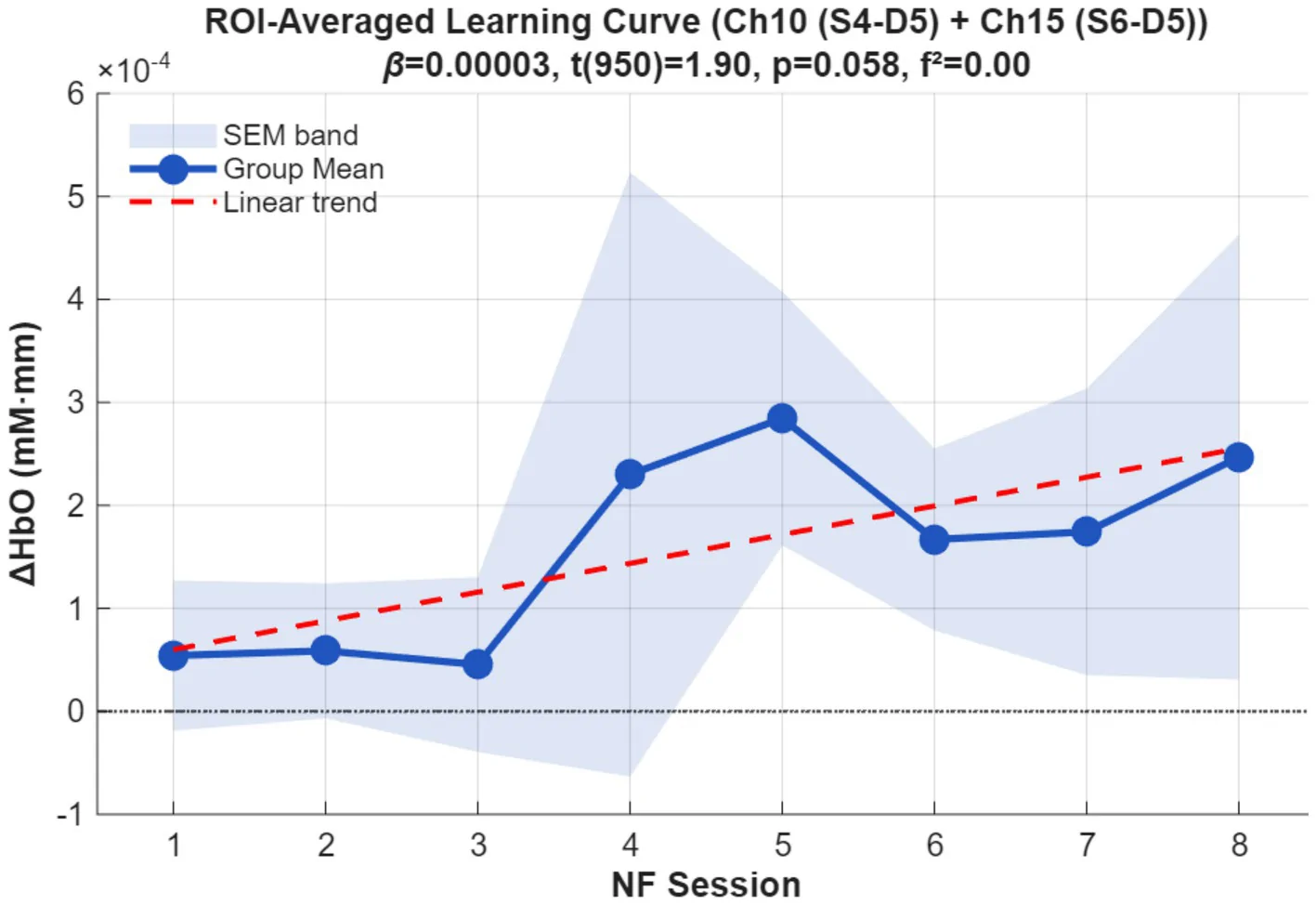
Channel-averaged (Ch10 + Ch15) dlPFC HbO learning curve across the eight NF sessions (*n* = 11). *β* = 0.00003, *t*(950) = 1.90, *p* = 0.058, *f*^2^ = 0.00. The shaded band represents ± SEM; the dashed red line shows the linear trend.

When Ch10 and Ch15 were averaged into a single dlPFC ROI, the group-mean ΔHbO learning curve (Figure 3) rose from approximately 0.55 × 10^−4^ mM·mm at Session 1 to a peak of approximately 2.85 × 10^−4^ mM·mm at Session 5, decreased by Sessions 6–7, and increased slightly at Session 8. A linear mixed-effects model fitted to this composite trajectory yielded *β* = 0.00003, *t*(950) = 1.90, *p* = 0.058, *f*^2^ ≈ 0.00, indicating a session-wise slope that approached, but did not reach, the conventional *α* = 0.05 significance threshold.

**Figure 4:**
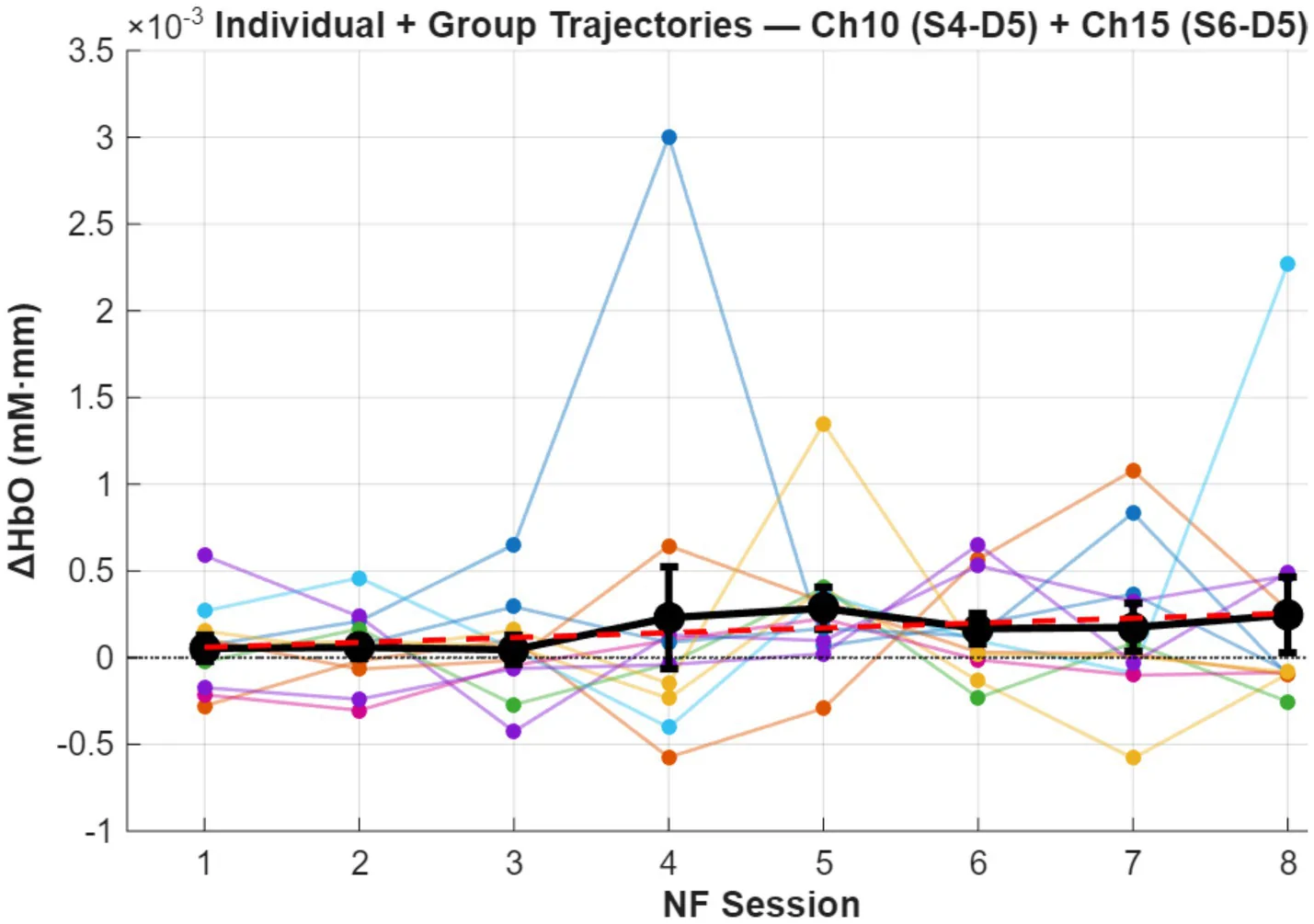
Individual participant HbO trajectories (thin coloured lines, *n* = 11) overlaid on the group-mean trajectory (thick black line ± SEM, red dashed = linear trend) across the eight NF sessions.

The individual trajectory plot demonstrated substantial between-participant heterogeneity. Several participants showed marked increases during later sessions, whereas others remained close to baseline or fluctuated across sessions throughout the intervention period (Figure 4).

### 3.3 Exploratory neural outcome: right dlPFC functional connectivity during NF

**Figure 5:**
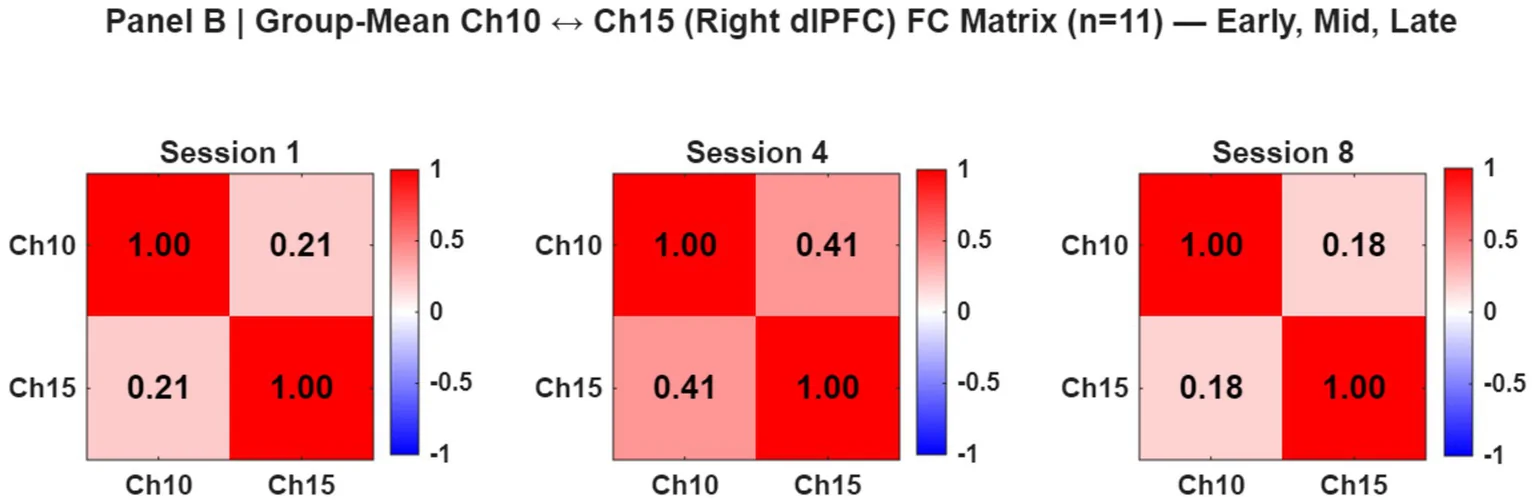
Group-mean Ch10↔Ch15 functional connectivity matrices (*n* = 11) at Session 1, Session 4, and Session 8 of the NF protocol.

As a secondary neural outcome of the trained dlPFC activity, Ch10↔Ch15 functional connectivity (FC) was analyzed across the eight sessions. The group-mean FC between Ch10 and Ch15 showed Fisher *z* values of 0.21 in Session 1, 0.41 at Session 4, and 0.18 in Session 8. These values indicate an increase in functional coupling during mid-training (Session 4), followed by a subsequent decline by the final session (Figure 5).

**Figure 6:**
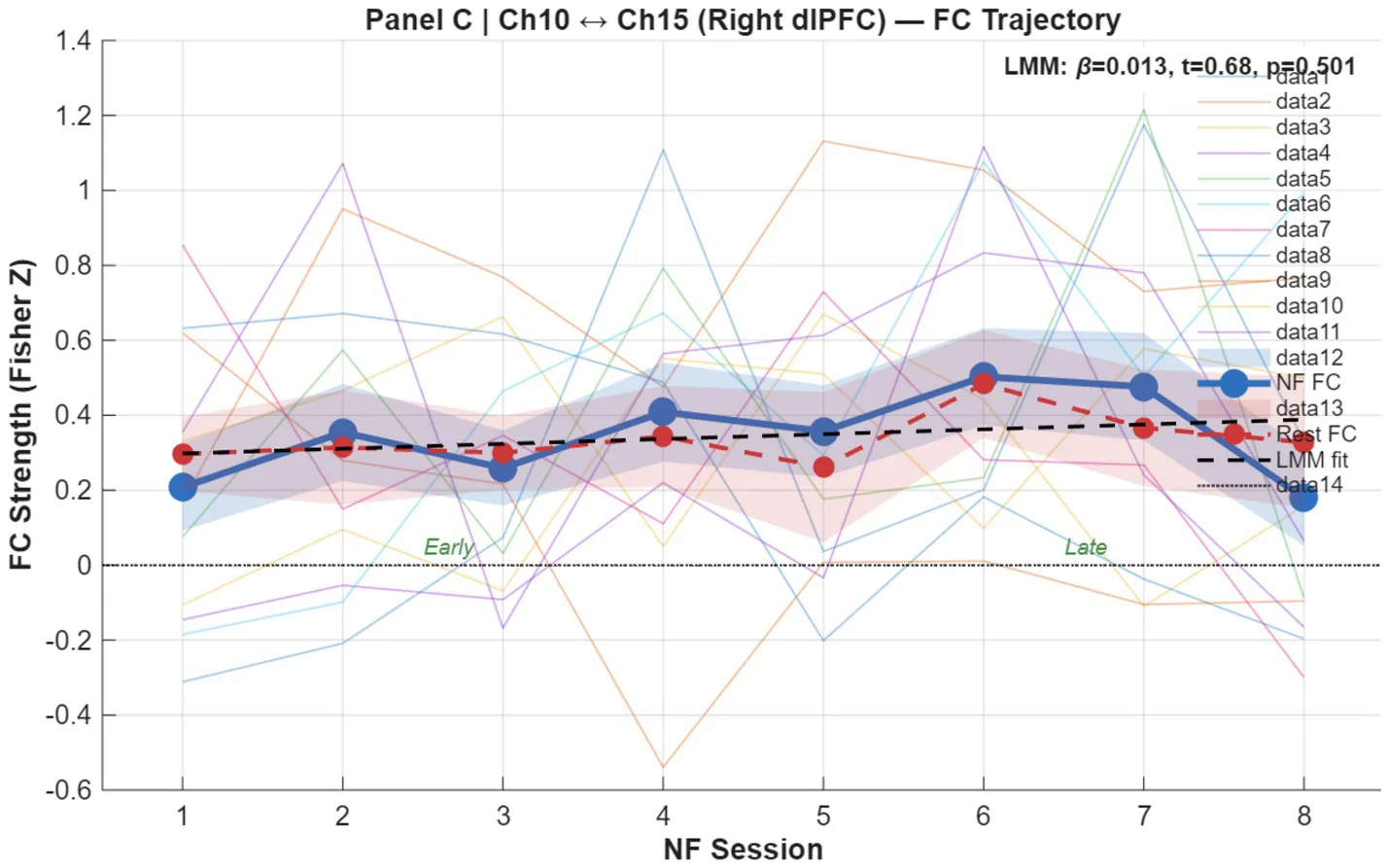
Ch10↔Ch15 individual FC trajectories across the eight NF sessions (*n* = 11). Thin colored lines represent individual participants; thick blue line shows NF-condition FC (*mean* ± SEM); the thick red dashed line represents resting-state FC; and the black dashed line shows the linear mixed-model fit (*β* = 0.013, *t* = 0.68, *p* = 0.501).

Individual connectivity trajectories showed a similar nonlinear profile and no statistically significant linear mixed-effects model slope (*β* = 0.013, *t* = 0.68, *p* = 0.501). Changes from Session 1 to Session 8 were heterogeneous across individuals, with some participants showing stronger end-of-training connectivity and others showing a reduction (Figure 6).

### 3.4 Behavioral pre–post outcomes

The *N*-back task was administered across three cognitive load conditions: 0-back, 1-back, and 2-back, with accuracy and mean reaction time (RT) recorded at each level.

Post-NF accuracy increased across all three *N*-back conditions relative to pre-NF performance. In the 0-back, 1-back, and 2-back conditions, mean accuracy increased by +0.152, +0.151, and +0.139, respectively, from pre- to post-assessment. None of the accuracy comparisons survived Bonferroni correction, although there was a consistent positive trend across all levels with moderate-to-large effect sizes.

Post-NF mean RT increased across all three *N*-back levels. The co-occurrence of improved accuracy alongside increased RT across all three levels may reflect a speed-accuracy tradeoff, whereby participants prioritized response accuracy over speed at post-assessment.

In the Stroop task, accuracy in the congruent condition showed minimal pre–post change, increasing from 0.821 to 0.850, with a negligible effect size. Congruent RT similarly showed minimal pre- to post-intervention change. In contrast, incongruent-trial accuracy improved substantially with an effect size of *d* = 0.700, indicating a large effect size. This represents the primary behavioral Stroop finding and is consistent with improved inhibitory control and conflict resolution following right dlPFC-targeted neurofeedback training.

The confidence intervals for all behavioral tasks were estimated using the Hodges–Lehmann estimator, accounting for the nonparametric distribution of the data.

**Figure 7:**
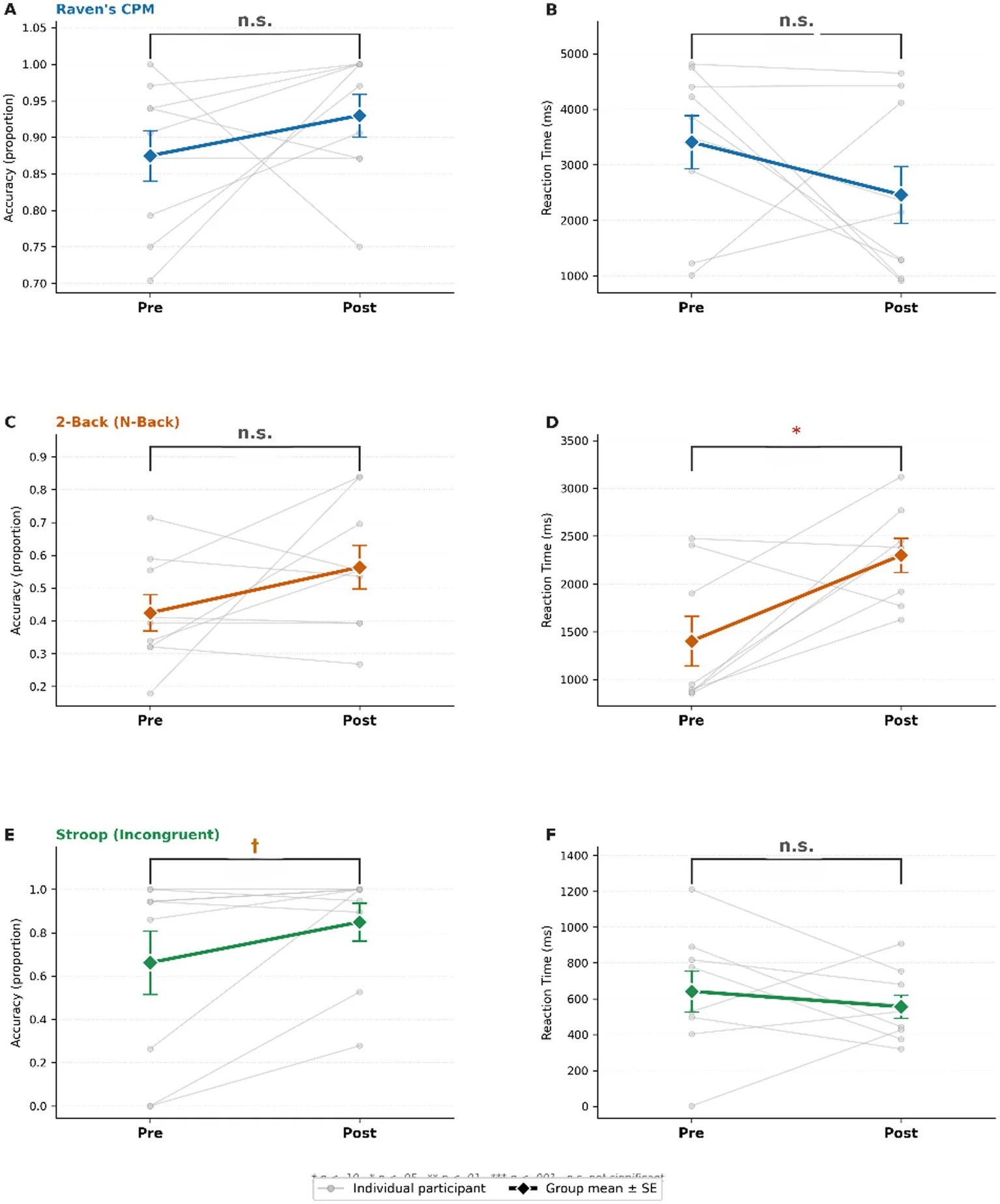
Pre–post group means (±SE) for the behavioral outcomes across the three executive functions. N-back at 2-back accuracy and RT, Stroop (incongruent) accuracy and RT, CPM (raw score and RT). The left panel for all tasks shows a pre–post shift in accuracy, and the right panel shows a pre-post shift in RT. n.s. = uncorrected non-significant.

Raven’s CPM showed a marginal increase in accuracy with a minimal change, where the effect size was negligible effect size, whereas mean RT decreased from pre- to post-intervention with a medium effect size (Table 1, Figure 7).

**Table 1:** Pre–post behavioral scores, mean (SD) for N-back, accuracy, and RT at all three levels, Stroop incongruent task, accuracy, and RT, and CPM, accuracy, and RT.

Measure	Pre (Mean ± SD)	Post (Mean ± SD)	Change	Rank-biserial *r*	p_uncorrected_	p_Bonferroni_	Δ Mean [95% CI]
N-back 0-back accuracy	0.656 (0.315)	0.808 (0.240)	+0.152	0.487	0.109	1.000	+0.152 [−0.082, +0.387]
N-back 1-back accuracy	0.498 (0.152)	0.649 (0.210)	+0.151	0.653	0.055	0.328	+0.151 [−0.014, +0.316]
N-back 2-back accuracy	0.436 (0.199)	0.575 (0.165)	+0.139	0.373	0.109	1.000	+0.139 [−0.065, +0.343]
N-back 0-back RT (ms)	1,097.1 (655.6)	2,168.7 (381.5)	+1,071.6	0.743	0.039	0.234	+1,071.6 [+324.9, +1,818.2]
N-back 1-back RT (ms)	1,381.2 (704.3)	2,397.2 (480.8)	+1,016.0	0.693	0.055	0.328	+1,016.0 [+104.0, +1,928.0]
N-back 2-back RT (ms)	1,404.1 (730.1)	2,299.5 (507.8)	+895.4	0.743	0.039	0.234	+895.4 [+172.8, +1,618.0]
Stroop incongruent accuracy	0.664 (0.440)	0.850 (0.264)	+0.185	0.72	0.031	0.125	+0.185 [−0.025, +0.400]
Stroop incongruent RT (ms)	721.64 (260.23)	555.94 (192.44)	−149.52	0.61	0.148	0.297	−149.52 [−395.71, +96.67]
CPM raw score	5.33 (3.20)	6.00 (2.96)	+0.67	0.11	0.738	1.000	+0.67 [−1.72, +3.04]
CPM RT (ms)	3,272.5 (1,540.8)	2,458.1 (1,540.2)	−814.34	0.38	0.250	0.500	−814.34 [−2,490.9, +862.2]

The change in scores, effect size, CI, p_uncorrected_, and p_Bonferroni_ are summarized for the scores, along with Bonferroni correction, which was non-significant for all the tasks.

A moderate-to-strong positive correlation was observed between right dlPFC HbO change and Stroop incongruent-trial accuracy improvement (*ρ* = +0.679, *p* = 0.094). No association was found for *N*-back 2-back accuracy (*ρ* = 0.000, *p* = 1.000), whereas *N*-back 2-back RT showed a moderate negative trend (*ρ* = −0.464, *p* = 0.294). Raven’s CPM accuracy showed no meaningful relationship (*ρ* = −0.107, *p* = 0.819). Overall, the pattern suggested a task-specific association, strongest for inhibitory control (Table 2).

**Table 2:** Spearman correlation analysis of targeted dlPFC changes between pre- and post-assessment and the behavioral outcomes, which are measures of stroop accuracy, N-back 2-back RT, and CPM accuracy.

Outcome	*ρ* (rho)	*p*-value	Interpretation
Stroop Incongruent accuracy	+0.679	0.094	Moderate-to-strong association
N-Back accuracy (2-back)	+0.000	1.000	No relation
N-back RT (2-back)	−0.464	0.294	Moderate negative relation
CPM accuracy	−0.107	0.819	No relation

## 4 Discussion

### 4.1 Summary of findings

To our knowledge, this pilot, proof-of-concept study represents the first report from the Indian subcontinent on an fNIRS-based neurofeedback (NF) protocol targeting the dorsolateral prefrontal cortex (dlPFC) in children who are first-degree relatives of individuals with substance use disorders and/or severe mental illness.

All 11 children recruited for the study completed the eight-session fNIRS-NF protocol targeting two right dlPFC channels, Ch10 and Ch15, without any observable adverse events or invalid sessions. The results showed: (i) a positive but statistically nonsignificant session-wise HbO trend in both target channels at the ROI level (*p* = 0.058); (ii) a nonmonotonic, nonsignificant functional connectivity pattern between Ch10 and Ch15, which rose from Session 1 to Session 4 before declining by Session 8 (*p* = 0.501); (iii) numerically higher accuracy across all three *N*-back load levels and Stroop incongruent-trial accuracy (*r* = 0.72), alongside numerically increased (slower) *N*-back reaction times across all three load levels—none of which survived Bonferroni correction; and (iv) an exploratory, underpowered positive association between pre–post neural change and Stroop incongruent accuracy improvement.

Overall, these findings demonstrate the feasibility of this intensive fNIRS-NF protocol in preadolescent children at high familial risk for psychiatric disorders and substance misuse. The changes in neural and behavioral measures were consistent with the expected effects of right-dlPFC-targeted neurofeedback, although hemodynamic and behavioral trends, while nearing conventional significance thresholds, did not achieve statistical significance due to limited statistical power in this pilot sample (*n* = 11).

### 4.2 Neurofeedback learning in right dlPFC

The primary neural finding demonstrated a positive, directionally consistent, but statistically nonsignificant upregulation of right dlPFC ΔHbO across the eight sessions, especially when combining Ch10 and Ch15 into a single region of interest. The group-level fixed-effect slope was positive and approached significance (*p* = 0.058). This suggests that participants demonstrated the capacity to enhance the target hemodynamic signal with repeated feedback sessions, although a sham-controlled trial is necessary to definitively confirm true self-regulation. This pattern indicates that the neurofeedback task was not merely tolerated, but also actively learned to a measurable extent. Although a rigorous frequency filter is used, because short-separation channels were not used (Section 5.1), some of this HbO trend may partly reflect superficial or systemic physiological signals. This learning is vital for any clinical or developmental use of fNIRS neurofeedback. In a similar pilot study involving 15 healthy adults using an eight-session fNIRS–NF protocol targeting the dlPFC, researchers observed increased dlPFC activity during neurofeedback. They also noted an increase in connectivity between the posterior cingulate and both the dlPFC and the striatum from Session 1 to Session 8 (Godet et al., 2024).

Individual trajectories showed considerable variability among participants rather than a consistent effect across the group. Some participants displayed strong upward trends in later sessions, while others remained near baseline or experienced varying patterns. This variability may reflect differences in attention, motivation, strategy development, fatigue, or baseline executive function. The nonlinear shape of this trajectory, which includes a peak at Session 5 followed by a decline and recovery, aligns with the session-to-session variability observed in other multisession fNIRS–NF studies. It may reflect the well-known impact of fatigue, motivation, and changes in attention on neurofeedback learning curves, particularly in children.

### 4.3 Functional connectivity dynamics: a nonmonotonic trajectory

The Ch10↔Ch15 FC trajectory observed here (Figure 5), rising from *r* = 0.21 (Session 1) to *r* = 0.41 (Session 4) before declining to *r* = 0.18 (Session 8), contrasts with the monotonic increase in connectivity reported in the eight-session adult dlPFC–NF study discussed before (Godet et al., 2024). Though the previous study used a different method and metrics (fMRI-based), in which posterior cingulate-dlPFC connectivity was observed to be higher in Session 8 than in session 1. Importantly, the present analysis focused on intra-regional connectivity between two right-dlPFC channels rather than on connectivity between the dlPFC and a distant hub region as in earlier studies. Intra-regional and inter-regional connectivity may follow different paths during NF training. Intra-regional connectivity might show a temporary peak in the middle of training as a child starts to develop a self-regulation strategy. After that, there may be some normalization as the strategy becomes more automatic and requires less coordinated activity among nearby channels. Notably, these intra-regional connectivity findings align with the earlier learning curve that indicated an increase in HbO amplitude around Sessions 4–5, followed by a decrease in the later stages.

This pattern suggests that functional coupling within the right dlPFC is more sensitive to intermediate learning stages than to the overall amount of training. The trajectory from Session 1 to Session 8 provides critical insight into individual-level dynamics (Figure 6). Some participants clearly increased FC over time, whereas others demonstrated a marked reduction. This divergence indicates that NF-induced FC changes are not merely a uniform group-level phenomenon but may reflect distinct individual regulatory trajectories. This finding aligns with a broader observation in the fNIRS–NF literature that connectivity changes after training are often less uniform across individuals than channel-specific activation magnitudes (Godet et al., 2024; Zeng et al., 2025). The key insight from these findings is the co-occurrence of directional hemodynamic learning with heterogeneous local network adaptations. The NF results demonstrate that participants could upregulate right dlPFC HbO across repeated sessions. Meanwhile, the FC results indicate that this regulation was characterized by dynamic, non-monotonic intra-regional coupling rather than a simple monotonic increase. Together, these findings suggest that the intervention fostered functional organization within the target region, though the specific pattern of reorganization varied across individuals. The target area became more consistently activated over time, local FC pattern did not follow a strictly linear path. This implies that regional hemodynamic regulation and intra-cortical network integration may operate along distinct developmental and temporal trajectories.

### 4.4 Pre–post behavioral changes

Improvement across all three N-back levels, along with a robust increase in Stroop incongruent-trial accuracy (*d* = 0.70), are consistent with the hypothesized benefits of right-dlPFC-targeted NF for working memory and inhibitory control. However, *N*-back reaction time numerically increased at all three levels (*r* = 0.74). The Stroop accuracy gain is particularly comparable to findings from a prior fNIRS–NF study, which showed a training-induced enhancement in Stroop performance (Zeng et al., 2025). Furthermore, the magnitude of the *N*-back effect sizes is comparable to those reported for significant working memory gains in other sham-controlled adult studies (Li et al., 2023). None of these effects survived Bonferroni correction given the pilot sample size of 11. The observed effect-size estimates provide a rigorous empirical basis for powering a future confirmatory randomized controlled trial.

The exploratory Spearman correlation findings suggest that right dlPFC HbO change may be more closely associated with inhibitory control, as indicated by Stroop task results. The Stroop incongruent-trial association (*ρ* = +0.679, *p* = 0.094) was the strongest behavioral marker and is consistent with the established role of the right dlPFC in response inhibition. The absence of associations with *N*-back accuracy and Raven’s CPM accuracy may reflect broader cognitive demands extending beyond the targeted ROI, as well as the small sample size. The RT negative correlation with 2-back RT (*ρ* = −0.464, *p* = 0.294) is directionally consistent with theoretical models, but underpowered. Because *N*-back RT increased from pre- to postintervention on average (Table 1), this negative correlation suggests that greater neural upregulation may be associated with a more attenuated RT slowing rather than with absolute response acceleration.

### 4.5 Limitations and future direction

Given the preliminary, proof-of-concept nature of this study, the sample size (*n* = 11) was justified on feasibility grounds rather than via an *a priori* power calculation. Most fundamentally, the absence of a sham-NF or active control condition precludes definitive attribution of the observed neural and behavioral changes specifically to the NF intervention itself, as opposed to nonspecific effects of repeated recording, sustained experimenter attention, practice, or maturation over the course of the study.

The target channels (Ch10, Ch15) defining the right dlPFC ROI used for the NF analyses represent only a subset of the 16-channel montage covering the bilateral dlPFC. Left-hemispheric and other right-hemispheric channels were not analyzed in the present report, and it remains possible that NF-induced changes were more pronounced in regions not examined here. In addition, short-separation channels were not included in this montage, so frontal HbO signals may be influenced by superficial or systemic physiological signals, and could not be regressed out. Finally, a fixed DPF of 6.0 was applied uniformly across the 6–12-year age range rather than an age-adjusted value, which may introduce minor inaccuracies into the quantification of absolute chromophore concentration, particularly for the youngest participants.

The immediate next course of action would be to repeat the study in a large, scalable, sham-controlled setting, so we can obtain a confirmatory study that tests the correlation between individual NF regulation success and behavioral improvement, powered to detect effects of the magnitude. Furthermore, structured, age-appropriate mental strategies employed during the NF should be systematically recorded at each session to determine whether the type of strategy or its consistency moderates hemodynamic self-regulation in children, as demonstrated in adults (Godet et al., 2024).

### 4.6 Conclusion

This fNIRS-based NF proof-of-concept study in an Indian pediatric at-risk population demonstrated high feasibility and tolerability: all 11 participants completed the eight-session protocol without adverse events. The ROI-averaged HbO trajectory showed a positive but statistically nonsignificant trend, approaching but not reaching the conventional threshold at the ROI level (*p* = 0.058), whereas intra-regional functional connectivity showed no significant linear trend (*p* = 0.501). Behavioral measures showed numerically higher post-intervention scores that did not survive Bonferroni correction. Notably, task-evoked right-dlPFC HbO during the Raven’s CPM and Stroop tasks shifted from predominantly negative pre-intervention values toward values closer to zero or positive post-intervention. These findings were directionally consistent across neural and behavioral measures, establishing the feasibility and preliminary direction of effect for fNIRS–NF in this novel population while providing effect-size estimates to inform the design of a larger, sham-controlled confirmatory trial. A large-scale study with a sham-controlled design is needed to confirm changes at neural and behavioral levels due to NF. This could serve as a starting point for non-pharmacological intervention studies to support the development of executive function in children at elevated risk.

## Data Availability

The datasets presented in this article are not readily available because the data under the PARAM cohort are subject to data governance and confidentiality agreement under which ethical approval is granted. De-identified data are available from the corresponding author upon reasonable request, subject to approval by the Institutional Ethics Committee, AIIMS Kalyani, in accordance with the ICMR data governance guidelines and the data-sharing agreement. Requests to access the datasets should be directed to Aniruddha Basu, aniruddha.psy@aiimskalyani.edu.in.
